# Iridoschisis: a case report and literature review

**DOI:** 10.1186/s12886-017-0418-2

**Published:** 2017-03-14

**Authors:** Yiyi Chen, Yiyong Qian, Peirong Lu

**Affiliations:** Department of Ophthalmology, The First Affiliated Hospital of Soochow University, 188 Shizi Street, Suzhou, 215006 People’s Republic of China

**Keywords:** Iridoschisis, Iris degeneration, Cataract, Phacoemulsification, Glaucoma

## Abstract

**Background:**

Iridoschisis is a rare condition that is characterized by the separation of the iris stroma into layers, with portions of the anterior layer floating freely in the aqueous humour. Here, we report three cases of iridoschisis that were complicated by either a cataract or glaucoma. Based on these cases, we speculate that the scope of iridoschisis has a rare association with intraocular pressure and the loss of corneal endothelial cells after surgery, which is mainly due to the surgery and not iridocorneal mechanical contact.

**Case presentation:**

We report three cases of iridoschisis, two of which were complicated by cataracts and the third by glaucoma. Patient 1 was a 69-year-old man with bilateral iridoschisis complicated by a cataract but not glaucoma, even though the entire anterior layer of the iris stroma in the right eye presented as white atrophic strands. To prevent the detached iris fibrils from invading the phacoemulsification tip and the irrigation/aspiration hand piece port, the separated iris stroma that was floating freely in the aqueous humour was cut with scissors immediately before the cataract extraction. Patient 2 was an 87-year-old woman with iridoschisis complicated by a cataract in the right eye. We successfully performed cataract surgery on the right eye without a pupillary device. Patient 3 was a 66-year-old man who presented with increased intraocular pressure with bilateral iridoschisis. He was discharged and prescribed with a combination of four glaucoma drugs.

**Conclusions:**

Patients with iridoschisis should have continuous follow-up because complications may occur, and extra care from ophthalmologists is required.

## Background

Iridoschisis is a rare condition in which the stroma of the iris is cleaved into two or more layers, and atrophy causes the anterior portion to disintegrate into fibrils with free ends that float in the anterior chamber (AC) [1, 2]. The age of onset of iridoschisis is most often between 60 and 70 years of age, and this condition usually originates in the inferior quadrants of the iris [3]. Iridoschisis is often associated with cataracts, angle-closure glaucoma, trauma, syphilitic interstitial keratitis, and congenital abnormalities [2, 4–6]. Patients with iridoschisis have experienced either abnormal intraocular pressure (IOP) or endothelial cell loss. However, in this case report, we suggest that the scope of the iris iridoschisis was not an important factor in the IOP and that the corneal endothelial cell loss after surgery was generally ascribed to the procedures during the surgery. To the best of our knowledge, this suggestion has not been previously reported.

## Case presentation

**Case 1** involved a 69-year-old male who presented with one month of blurred vision and an exacerbation of this blurred vision for the preceding ten days in the right eye (OD). The patient had no history of ocular trauma or heritable ocular disease. He had been suffering from hypertension for 15 years and was receiving antihypertensive treatment.

**Fig. 1:**
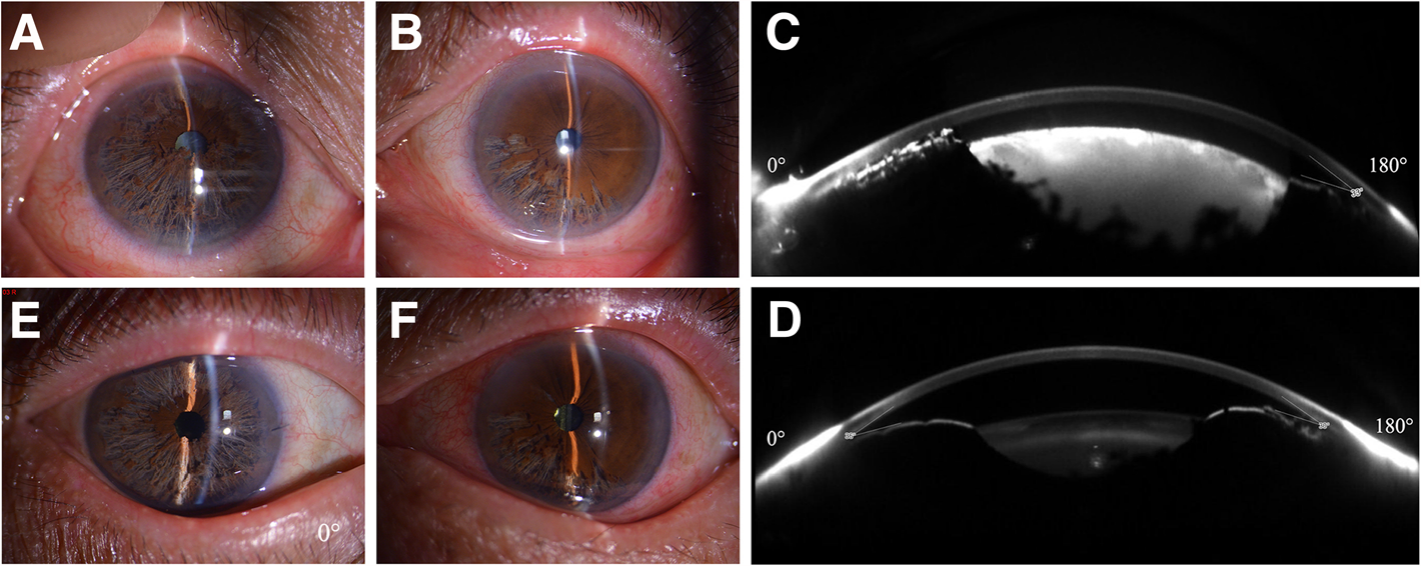
Slit-lamp photographs and Scheimpflug images by Sirius of case 1. **a** In the *right eye*, four quadrants of “shredded wheat” appearance were observed. **b** In the *left eye*, an inferior-nasal iris defect with a curl of the anterior layer of the iris was observed. **c** Scheimpflug images (before operation) of the right eye, the local anterior chamber was remarkably shallow (CCT + AD = 0.515 + 1.60 = 2.11 mm), and the iridocorneal angle at the 180° direction was 33°. **d** In the *left eye*, the anterior chamber was normal (CCT + AD = 0.509 + 1.97 = 2.48 mm), and the angle at the 180° direction was 30°. **e** Slit-lamp photographs of the anterior segment one month postoperatively. Four quadrants of “shredded wheat” appearance similar to preoperative status and an AC of normal depth were observed in the right eye. **f** In the *left eye*, an inferior-nasal iris defect with a curl of the anterior layer of the iris was observed

The result of a rapid plasma reagin (RPR) test for syphilis was negative. A clinical examination showed that the preoperative uncorrected visual acuities (UCVA) were hand movement OD and 20/40 for the left eye (OS). The IOP in each eye was 13 mmHg. Slit-lamp biomicroscopy showed that the anterior layer of the iris stroma (OD) was divided into a loose mixture of numerous pigmented and white atrophic strands, which ran in all directions and thus presented an interlaced pattern. The distal ends of the fibrils were attached to the ciliary portion and were floating freely in the aqueous humour (Fig. 1a). The underlying iris pigment epithelium appeared imperfect, which indicated the transillumination defect, and the exfoliating iris pigment epithelium accumulating in the AC could also be observed. The right eye showed a mature cataract that hindered visualization of the fundus and evaluation of any optic disc alterations. A slit-lamp examination of the left eye revealed inferior-nasal iridoschisis, which presented from the 5 to 9 o’clock positions and had a normal AC depth (Fig. 1b). Scheimpflug image by Sirius (Costruzione Strumenti Oftalmici, Florence, Italy) showed that the local AC was extremely shallow in the right eye (Figs. 1c, d). The endothelial cell counts were 3,453 cells/mm^2^ OD and 3,738 cells/mm^2^ OS.

Phacoemulsification (PHACO) was first performed on the right eye and was then conducted on the left eye one month later. During surgery, the freely floating iris fibrils were cut with Vannas capsulotomy scissors. A foldable hydrophobic acrylic IQ intraocular lens (IOL) (Acry-Sof® SN60WF, Alcon Laboratories Inc.) was safely implanted in the capsular bag. Postoperative examinations were conducted at 1, 7, 30, and 60 days following the procedure.

One day after the surgery on the right eye, the UCVA of the right eye was FC/30 cm, and the IOP was 13 mmHg. A slit-lamp examination showed moderate ocular hyperaemia and corneal oedema. The AC depth was normal, and the pupil was round and undamaged. One week after surgery, the UCVA was 20/50 and the IOP was 15 mmHg. The ocular hyperaemia had disappeared, and there was mild corneal oedema that was improving. One month after surgery, the UCVA was 20/40 and the IOP was 17 mmHg (Fig. 1e). Two months after surgery, the UCVA was 20/30, the IOP was 17 mmHg, and the endothelial cell count was 1,085 cells/mm^2^.

One month after the surgery on the right eye, PHACO was performed on the left eye without additional treatment to the iris. One day after the surgery on the left eye, the UCVA in that eye was 20/32, and the IOP was 16 mmHg. A slit-lamp examination showed no obvious postoperative reaction. One week after surgery, the UCVA was 20/30 and the IOP was 17 mmHg. One month after surgery, the UCVA was 20/25 and the IOP was 16 mmHg (Fig. 1f). The endothelial cell count was 2,630 cells/mm^2^ at that time. Two months after surgery, the UCVA was 20/25, the IOP was 14 mmHg, and the endothelial cell count was 3,618 cells/mm^2^. Although the postoperative corneal endothelial cell density remains normal, the coefficient of variation > 30.

**Case 2** involved an 87-year-old female who presented with blurred vision in both eyes for the preceding two years. The patient had suffered from diabetes mellitus for four years, had an allergy to penicillin, had a history of hypertension, and was on antihypertensive and antidiabetic treatments. She had received cataract surgery on her left eye two years earlier and had no history of ocular trauma or heritable ocular disease.

**Fig. 2:**
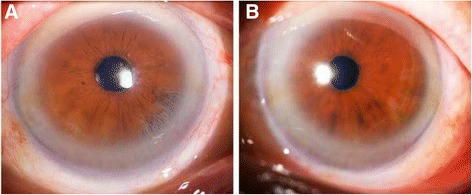
Slit-lamp photographs of the patient in case 2’s anterior segment. **a** In the *right eye*, the inferior-nasal iris defect of the iris was observed. **b** In the *left eye*, the inferior-temporal iris atrophy was observed

The result of a RPR test for syphilis was negative. A clinical examination showed that the preoperative best corrected visual acuities (BCVAs) were 20/200 OD and 20/200 OS. The IOPs were 12 mmHg OD and 10 mmHg OS. Slit-lamp biomicroscopy of the right eye showed a “shredded-wheat” appearance in the nasal quadrants (from the 4 to 6 o’clock positions). The pupil of this eye was round and had a normal reaction to light. The lens showed a mature cataract, which hindered visualization of the fundus and evaluation of any optic disc alterations. The AC was normal, and the angle was open. The endothelial cell count was 3,068 cells/mm^2^. Slit-lamp biomicroscopy of the left eye showed iris atrophy in the inferior temporal quadrants (Fig. 2b). The IOL of the left eye was in position. Because the patient in Case 2 had a right eye that was similar to the left eye of the patient in Case 1 and considering the limited extent of the iridoschisis, conventional PHACO was performed on the patient in Case 2 to remove the cataract, and a single-piece acrylic intraocular lens (Zeiss® CT SPHERIS 209 M, Zeiss) of 16.0 D was then safely implanted in the capsular bag.

One day after surgery on the right eye, the UCVA was 20/80 and the IOP was 10 mmHg. Slit-lamp biomicroscopy showed moderate corneal oedema, an AC of normal depth, and some fibrillary materials. The pupil was round and undamaged. One week after surgery, the UCVA was 20/63 and the IOP was 13 mmHg. The ocular hyperaemia had disappeared and the mild corneal oedema was improving. One month after surgery, the UCVA was 20/50 and the IOP was 13 mmHg. The endothelial cell count was 1,456 cells/mm^2^. A slit-lamp examination of the right eye revealed inferior-nasal iridoschisis that was presented from the 4 to 6 o’clock positions and had an AC of normal depth (Fig. 2a).

**Case 3** involved a 66-year-old male who was referred to our clinic because of visual loss with congestion and intermittent pain in the left eye for the preceding six months. The patient had no history of ocular trauma or heritable ocular disease.

**Fig. 3:**
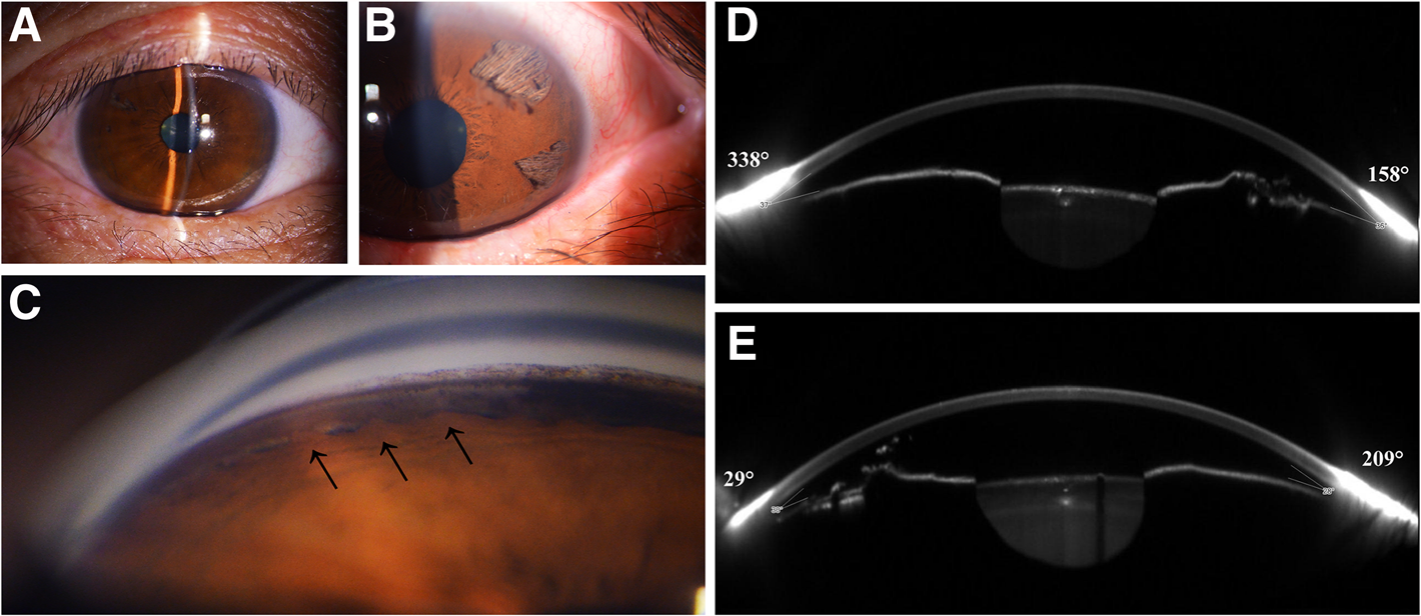
Slit-lamp photographs, gonioscopy and the Scheimpflug images by Sirius of the patient in case 3. **a** In the *right eye*, a local rupture in the temporal quadrant of the iris was observed. **b** In the *left eye*, the temporal iris defect was observed. **c** Gonioscopy showed that the peripheral anterior chamber in the superior area of the right eye had discontinuous synechiae together with pigment deposition. **d** Scheimpflug image by Sirius of the *right eye* showed that the anterior chamber was normal (CCT + AD = 0.531 + 3.05 = 3.59 mm) and the iridocorneal angle at a 158° direction was 36°. **e** In the *left eye*, the anterior chamber was not shallow (CCT + AD = 0.516 + 2.66 = 3.17 mm), and the angle at a 29° direction was 30°

The result of a RPR test for syphilis was confirmed to be a false positive. The preoperative BCVAs were 20/125 OD and 20/50 OS. The IOPs were 22 mmHg OD and 35 mmHg OS. Slit-lamp examination revealed a local rupture in the temporal quadrant of the iris stroma of the right eye (Fig. 3a). In the left eye, the anterior layer was divided into a loose mixture in the temporal iris and the AC was of normal depth (Fig. 3b). A postmydriatic examination showed cortical opacity of the lens and dust turbidity of the vitreous body. Gonioscopy showed that the peripheral AC in the superior area of the right eye had a discontinuous synechia along together with pigment deposition (Fig. 3c). Scheimpflug images by Sirius showed that part of the peripheral AC was shallow and that the iridocorneal angle was still open in both eyes (Figs. 3d, e). The endothelial cell counts were 3,306 cells/mm^2^ OD and 3,011 cells/mm^2^ OS. The patient was diagnosed with secondary glaucoma with iridoschisis and received BAK-free travoprost 0.004% containing polyquaternium-1 (Travatan® preserved with POLYQUAD®, Alcon Laboratories, Fort Worth, TX, USA) once daily and brinzolamide (Azopt®, Alcon, Laboratories, Elkridge, MD), 2% carteolol (Mikelan®, China Dazhong pharmaceutical Co., Ltd, Tianjin, China), and alpha2-agonist (Alphagan®, Allergan, Inc., Irvine, CA) twice daily.

One month after admission, over which time the eye drops were used, the IOPs were 18 mmHg OD and 32 mmHg OS. The IOPs showed significant fluctuation with medical treatment. Notably, glaucoma surgery will be necessary for this patient in the future. The patient was advised to have frequent ophthalmologic examinations to monitor the IOPs in his eyes and to assess any progression of glaucomatous changes in both eyes.

## Discussion

Iridoschisis is a rare condition that is characterized by partial cleavage of the iris. The anterior layer of the iris stroma, which is similar in appearance to seaweed, becomes curved and floats freely in the anterior chamber (AC). Some portions of the posterior layer become thin and atrophied and is similar in appearance to shredded wheat [7].

This condition was first described in 1922 by Schmitt, who presented a case with detachment of the anterior iris layer [8]. The term iridoschisis (iris splitting) was first introduced in 1945 by Lowenstein and Foster, who found a deep, parallel cleft between the anterior and posterior stromal layers of the iris [1]. Iridoschisis is most commonly considered to be an age-related atrophy; however, although its causative agent has not yet been identified, cataracts are often an attendant issue [5].

Cataract operations in patients with iridoschisis require special care. Several methods have been used to stabilize the iris fibres, such as flexible iris hooks, and the use of ophthalmic viscosurgical devices (OVDs) [9–11]. In our first case, after the injection of an OVD into the AC, the iris fibrils were cut by Vannas capsulotomy scissors to prevent the detached iris fibrils in the AC from invading the PHACO tip and the irrigation/aspiration hand piece port. We successfully performed cataract surgery on the right eye without a pupillary device.

In both Cases 1 and 2, we minimized any iris trauma by conducting the operation without an extra pupil-support device. In Case 1, when the UCVA of the left eye was 20/40, we advised the patient to receive cataract surgery sooner than usual with a twofold aim: to prevent blindness caused by the cataract and to avoid additional damage to the ruptured iris tissue from further surgery [10]. One month after surgery, the UCVA of the left eye was 20/25, and a slit-lamp examination did not show any expansion of the scope of the iridoschisis. Based on previous experiences, mechanical restraint of the diseased portion of the iris can be useful for cataract surgery. From our observations, in some conditions, PHACO performed without pupillary devices can also achieve a satisfactory result (even in patients with a wide range of iridoschisis).

Another important aspect of iridoschisis is its frequent association with glaucoma. Glaucoma occurs in more than 50% of iridoschisis cases and is often classified as angle-closure glaucoma [12]. The IOP of Patient 1, who presented with a shredded wheat appearance in all four quadrants, was at a normal level. The IOP of Patient 3 showed significant fluctuations after medical treatment. Scheimpflug images by Sirius indicated that the iridocorneal angle was open in both eyes. Gonioscopy showed that the peripheral AC in the superior area of the right eye had discontinuous synechiae together with pigment deposition. We presumed that the shredded wheat fibres contributed to the angle crowing. However, we speculated that the high IOP was a result of an interaction between the shredded wheat fibres and other factors. In Case 3, the peripheral AC angle recession with accompanying pigment deposition was a prominent feature. Consequently, glaucoma surgery will be necessary in the future. Therefore, high IOP may be the joint effect of either split iris tissue, synechiae, or plateau iris configuration, which may be the main causes of primary angle-closure glaucoma in patients with iridoschisis.

Furthermore, iridoschisis was previously reported to be associated with corneal endothelial decompensation, which is an uncommon complication. However, several cases of corneal decompensation secondary to iridoschisis have been reported [13–15]. Mechanical iridocorneal contact was thought to cause corneal endothelial decompensation [14]. The preoperative corneal endothelial cell densities of our cases were within normal limits. However, in Case 1, the endothelial cell count was 1,085 cells/mm^2^ for OD and 3,618 cells/mm^2^ for OS two months after surgery. Due to the relatively long operation process required to remove the freely floating iris fibrils in the aqueous humour of the right eye, corneal oedema was observed in the early stage after surgery, and we found no iris/cornea contact with our postoperative slit-lamp microscope inspection. Therefore, surgery rather than iridocorneal mechanical contact was the main cause of the postoperative decrease in corneal endothelial cells.

The differential diagnosis for iridoschisis includes the neurocristopathy of the iris and cornea (Axenfeld-Reiger [AR] syndrome) and iridocorneal endothelial (ICE) syndrome. AR syndrome is a rare, non-progressive congenital anomaly that presents at birth. Bilateral changes have been found in both AR syndrome and iridoschisis. Moreover, AR syndrome is characterized by anterior displacement of Schwalbe’s line and hypoplasia of the iris stroma with filaments connected to an abnormal peripheral cornea. In contrast to ICE syndrome, bilateral onset and old-age onset are two important features of iridoschisis. ICE syndrome is more frequent in young adults, especially in females aged 30 to 40 years, and is usually unilaterally involved. The clinical features of ICE syndrome include corectopia or polyconic projection, areas of atrophy, full-thickness hole(s), and nodules in the iris, endothelial dystrophy, identification of ICE cells by confocal microscopy, corneal oedema, and peripheral anterior synechiae (PAS) of the AC angle. The iris tissue is usually atrophic rather than split and is characterized by marked iris atrophy and hole(s) formation. The iris tissue vanishes without previous signs due to tissue ischaemia [16]. By contrast, normal perfusion of blood vessels has been reported in patients with iridoschisis [17]. The iris stroma, which shows splitting instead of hole formation, is pronounced and similar to the observations in Scheimpflug image by Sirius. Indentation gonioscopy shows that the PAS extends to the Schwalbe’s line in patients with ICE. The pupil may be ectopic and displaced towards the PAS. Specular microscopy has also revealed abnormal corneal endothelial cells or so-called “ICE cells.” In these cases, the difference between iridoschisis and iris atrophy can be determined easily by ultrasound biomicroscope (UBM) and Scheimpflug image by Sirius. In patients with iridoschisis, as the separation of the iris stroma and loosening of the tissue occurs, the distance between the anterior and posterior layers increases and shows an irregular reflection [18]. A narrow AC and the discontinuous iris pigment epithelium can also be clearly detected. Furthermore, UBM is the optimal method for diagnosing plateau iris syndrome because it enables functional testing of both the iris and the ciliary body [19].

In summary, an iridoschisis diagnosis should made based on the results of UBM and Scheimpflug image by Sirius. With the use of UBM and Scheimpflug image, iridoschisis can be easily distinguished from other anomalous iris diseases.

## Conclusions

Iridoschisis is an age-related condition that is usually accompanied by cataracts, secondary glaucoma, or corneal endothelial decompensation. Patients with iridoschisis that is complicated by cataracts should receive cataract surgery sooner than usual to achieve better outcomes. High IOP in these patients may be due to the joint effect of multiple factors rather than only the scope of the iridoschisis. Finally, because surgery may cause a severe decrease in postoperative corneal endothelial cells and various complications, cataract operations in patients with iridoschisis require special care.

## Abbreviations

AC: Anterior chamber
AD: Anterior chamber depth
BCVA: Best corrected visual acuity
CCT: Central corneal thickness
I&A: Irrigation &aspiration
ICES: Iridocorneal endothelial syndrome
IOL: Intraocular lens
IOP: Intraocular pressure
OD: Right eye
OS: Left eye
OVD: Ophthalmic viscosurgical device
PAS: Peripheral anterior synechiaes
PHACO: Phacoemulsification
RPR: Rapid plasma reagin
UBM: Ultrasound biomicroscope
UCVA: Uncorrected visual acuity
